# Newborn screening for severe combined immunodeficiency: The results of the first pilot TREC and KREC study in Ukraine with involving of 10,350 neonates

**DOI:** 10.3389/fimmu.2022.999664

**Published:** 2022-09-15

**Authors:** Oksana Boyarchuk, Nataliia Yarema, Volodymyr Kravets, Oleksandra Shulhai, Ivanna Shymanska, Iryna Chornomydz, Tetyana Hariyan, Liubov Volianska, Maria Kinash, Halyna Makukh

**Affiliations:** ^1^ Department of Children's Diseases and Pediatric Surgery, I.Horbachevsky Ternopil National Medical University, Ternopil, Ukraine; ^2^ Department of the Research and Biotechnology of Scientific Medical Genetic Center "Leogene, LTD", Lviv, Ukraine; ^3^ Department of the Diagnostics of Hereditary Pathology, Institute of Hereditary Pathology of the Ukrainian National Academy of Medical Sciences, Lviv, Ukraine

**Keywords:** newborn screening, TREC, KREC, severe combined immunodeficiency, inborn errors of immunity

## Abstract

Severe combined immunodeficiency (SCID) is a group of inborn errors of immunity (IEI) characterized by severe T- and/or B-lymphopenia. At birth, there are usually no clinical signs of the disease, but in the first year of life, often in the first months the disease manifests with severe infections. Timely diagnosis and treatment play a crucial role in patient survival. In Ukraine, the expansion of hemostatic stem cell transplantation and the development of a registry of bone marrow donors in the last few years have created opportunities for early correction of IEI and improving the quality and life expectancy of children with SCID. For the first time in Ukraine, we initiated a pilot study on newborn screening for severe combined immunodeficiency and T-cell lymphopenia by determining T cell receptor excision circles (TRECs) and kappa-deleting recombination excision circles (KRECs). The analysis of TREC and KREC was performed by real-time polymerase chain reaction (RT-PCR) followed by analysis of melting curves in neonatal dry blood spots (DBS). The DBS samples were collected between May 2020 and January 2022. In total, 10,350 newborns were screened. Sixty-five blood DNA samples were used for control: 25 from patients with ataxia-telangiectasia, 37 - from patients with Nijmegen breakage syndrome, 1 – with X-linked agammaglobulinemia, 2 – with SCID (JAK3 deficiency and DCLRE1C deficiency). Retest from the first DBS was provided in 5.8% of patients. New sample test was needed in 73 (0.7%) of newborns. Referral to confirm or rule out the diagnosis was used in 3 cases, including one urgent abnormal value. CID (T^low^B+NK+) was confirmed in a patient with the urgent abnormal value. The results of a pilot study in Ukraine are compared to other studies (the referral rate 1: 3,450). Approbation of the method on DNA samples of children with ataxia-telangiectasia and Nijmegen syndrome showed a high sensitivity of TRECs (a total of 95.2% with cut-off 2000 copies per 10^6^ cells) for the detection of these diseases. Thus, the tested method has shown its effectiveness for the detection of T- and B-lymphopenia and can be used for implementation of newborn screening for SCID in Ukraine.

## Introduction

Severe Combined Immunodeficiency (SCID) is a genetically heterogeneous group of diseases that are accompanied by impaired T- and B-cell immune responses, leading to a combined dysregulation of cellular and humoral immunity (1). Patients with SCID are usually born with no clinical signs of the disease, but within the first year, and often within the first months of life in children with T-lymphocyte deficiency, and within the second half of the first year in children with certain defects in antibody production, the disease manifests as severe infections (2). Without proper diagnosis and timely immune-restoring treatments the children die in the first or second year of life. Hematopoietic stem cell transplantation (HSCT), enzyme replacement therapy for adenosine deaminase (ADA) deficiency, and gene therapy in some disease types are the only curative treatments today that allow not only to save life but also to ensure its quality (3). Studies of this disorders for the past 20 years show a considerably high chance of survival in those who underwent HSCT before 3.5 months of age, reaching up to 94% survival rate over 6 years (3, 4).

SCID affects approximately 1: 50,000-1: 100,000 newborns and only up to a third of the newly diagnosed cases have a family history (5, 6). Certain ethnic groups have a higher incidence of SCID because of founder mutations. For example, in Somalia ADA-SCID occurs 1: 5,000, while the DCLRE1C gene mutation (Artemis) in Navajo Americans is even more common, 1: 2,000 (6, 7). The rapid development of genetics contributes to the discovery of new mutations that cause inborn errors of immunity (IEI), including SCID (1).

Since SCID is a rare disease which is accompanied by life-threatening health problems, is not determined by standard clinical examinations, and has cutarive treatments, the effectiveness of which depends on the time of diagnosis, the need and appropriateness of screening has become evident (8, 9).

Neonatal screening, which was first introduced in the 1960s, now covers dozens of diseases and has reached great progress in Europe and worldwide in recent years (10). In 2008, newborn screening for severe combined immunodeficiency (SCID) was first introduced in Wisconsin, USA (11). Since then, newborn screening for SCID has implemented not only in all states of the USA, but also in many countries of Europe, the Middle East and Asia (12–18).

Of methods proposed for screening diagnosis of SCID, a DNA based technique has received prominence (8). It is a quantitative molecular genetic method of detecting T-cell receptor excision circles (TRECs), which are a by-product of T-cell differentiation in the thymus and thus can be used as a marker of T-lymphopenia (8, 11). The TREC analysis was developed by Douek et al., who demonstrated that TRECs are specific for naive T-cells and undergo decline either due to ageor infections such as HIV (19). In 2005, Puck and Chan proposed to use this technique for population-based SCID screening (20).

The TREC assay can only detect T-lymphopenia. However, there is another group of severe IEI associated with B-lymphocyte deficiency, including X-linked agammaglobulinemia (XLA, Bruton’s disease) and autosomal recessive hypogammaglobulinemia, which also lead to life-threatening conditions. In 2007, a method was developed based on the detection of kappa-deleting recombination excision circles (KRECs), DNA fragments formed during the maturation of B-cells in the bone marrow, using polimerase chain reaction (PCR) (21). Combining these two techniques allows to detect not only congenital T-cell defects, but also other forms of IEI, which can be missed by analysis of TREC only, in particular, late onset of ADA deficiency, Nijmegen breakage syndrome (NBS) and other conditions (22). The issue of compliance of KREC assay with the general principles of neonatal screening is currently being discussed.

However, as the results of screening programs shown, low levels of TRECs/KRECs are detected not only in SCID, but also in other immunodeficiencies and diseases that are characterized by low levels of T- and/or B-cells (23–25).

Newborn screening for severe T- and/or B-cell deficiencies is an important tool for the timely diagnosis of IEI (26, 27). Diagnosis and treatment of congenital defects of the immune system have made significant strides in many countries, including Ukraine. However, in a significant proportion of children with severe immune deficiency in the first year of life, this diagnosis is made posthumously. These congenital defects are underdiagnosed primarily because of the lack of opportunities for early diagnosis and low awareness of these pathologies in doctors and the other health care professionals (28, 29). In recent years in Ukraine, the expansion of HSCT and the development of a bone marrow donors registry have created opportunities for early correction of IEI and improving the quality and life expectancy of children with SCID. Therefore, the issue of early SCID detection using newborm screening becomes a pressing one.

## Materials and methods

In 2020, we initiated the project “A pilot study on newborn screening for primary immunodeficiencies using TREC/KREC assay to identify T- and B-lymphopenia”, which received support from the Ministry of Health of Ukraine. The study was performed according to the 1975 Declaration of Helsinki (as revised in 2000), and approved by the I.Horbachevsky Ternopil National Medical University Ethics Committee (Minutes № 55 from November 4, 2019).

In addition to the 4 diseases (phenylketonuria, cystic fibrosis, congenital adrenal hyperplasia, congenital hypothyroidism) of the newborn screening panel that are already being tested for in Ukraine, we included a screening for primary immunodeficiencies using TREC/KREC assay. The additional dried blood spot (DBS) for the SCID screening was collected between May 2020 and January 2022 (21 months in total). The pilot study covered the Ternopil region in western Ukraine. A total of 15 maternity hospitals in the region were involved in the study, including two maternity hospitals in the region capital, which had the largest numbers of births (5591 DBS). In addition, patients from the neonatal intensive care units of the neighboring Ivano-Frankivsk and Lviv regions were included the study. As of 01.01.2021, the population of Ternopil region was 1,030,600 inhabitants.

The TREC/KREC determination was performed at the Scientific Medical Genetic Center LeoGENE, LTD, Lviv, Ukraine. The other project partners were the Institute of Hereditary Pathology of the Ukrainian National Academy of Medical Sciences, Lviv, Ukraine; Western-Ukrainian Specialized Children’s Medical Center, Lviv, Ukraine; and Ivano-Frankivsk Regional Children’s Hospital, Ivano-Frankivsk, Ukraine.

We organized several information meetings for pediatricians, neonatologists, nurses, during which we provided information about the screening, its purpose and features. Neonatologists and nurses informed parents about the project and parental consent was obtained for each newborn.

Heel prick blood spot tests were performed mainly on the third day post-partum as part of the national NBS program. Blood samples were blotted on a blank disc of filter paper, which carries a unique number and information about the newborn. Blood spots were dried at a room temperature for at least 3 hours, protected from direct sunlight and stored at a temperature of +2 to +8°C.

Data on all DBS were entered into a computer database shared with the center where the analysis was performed. The database included key characteristics of the newborns, such as birth date, date of sample collection, sex, birth weight (BW), and gestational age (GA). According to the WHO definition, newborns with GA ≥38 weeks were defined as born at term; GA ≥32–37 weeks – as moderate preterm, GA ≥28–32 weeks - very preterm; <28 weeks - extremely preterm (30).

The analysis of TREC and KREC was performed by real-time polymerase chain reaction (RT-PCR) followed by analysis of melting curves in neonatal DBS. Due to the disruptions caused by the COVID-19 pandemic, it was not feasible to obtain commercial kits for screening. This compelled the Scientific Medical Genetic Center LeoGENE to adapt their methodology and develop a proprietary testing approach.

At the first stage of the pilot study, when logistical issues were agreed upon and the methodology was worked out, the time between the blood sampling and obtaining the newborn screening results was 3-5 weeks. At the second stage, we managed to reduce this time to 10 days.

### Sample processing and real –time PCR

Isolation and purification of DNA from DBS on filter paper was carried out using a kit for the isolation of nucleic acids DNA-SorbB (AmplySens, RF). The DNA sample was dissolved in 75 μl of TE buffer and the concentration and optical characteristics were determined using the DENOVIX instrument.

The quantity of TREC and KREC molecules was analyzed by RT-PCR. Primer sequences used to amplify the sequences of TREC were (F: 5’-CCATGCTGACACCTCTGGT-3’, R: 5’-TCGTGAGAACGGTGAATGAAG-3’), KREC (F: 5’-TCAGCGCCCATTACGTTTCT-3’, R: 5` - GTGAGGGACAC GCAGCC-3’) and the albumin gene as an internal control (F: 5’-TGAACAGGCGACCATGCTT-3’, R: 5’-CTCTCCTTCTCAGAAAGTGTGCATAT-3’) according to the protocol (31). RT-PCR reactions were prepared for each of the three primer pairs and contained 6 µl of dd water, 1.5 µl of 5xHOT FIREPol EvaGreen Mix Plus (Solis, BioDyne, Estonia), 0.3 µl of primers and 2 µl of DNA samples (10-25 ng/µl).

Plasmids with a known number of TREC and KREC copies were used as standards in RT-PCR. Deionized water was used as a no template control. DNA sample of patient with NBS and low number of TRECs and KRECs was used as a positive control. Real-time PCR was performed with the following parameters: polymerase activation (50°C, 2 min), initial denaturation (95°C, 10 min) and 50 cycles of following: denaturation (at 95°C, 15 sec), annealing and extension (at 60°C, 60 sec). The RT-PCR was carried out on CFX96 Touch Real-Time PCR Detection System, Bio-Rad, USA. The Ct of positive control is 3-5 cycles above the majority of DNA samples we analyzed (Ct = 30 - 33). The RT-PCR amplification curves of DNA samples with low TRECs and/or KRECs were similar to NTC curves.

Due to the low copy number of TRECs and KRECs, compared to nuclear DNA genes, an additional analysis of PCR products by the melting method was performed to increase the specificity of the method. Analysis of melting curves was performed at a temperature from 50°C to 90°C with a step of 0.5°C (melting analysis). This technique is based on the dependence between the melting temperature of DNA fragments to their primary structure. This additional step was implemented to differentiate negative results from unspecific PCR product.

We used as a comparative method RT-PCR with fluorescent probes for three targets: albumin gene, TREC and KREC. RT-PCR reaction was prepared for three different primer pairs and contained 2.5 µl of dd water, 5µl of 2xMaxima Probe qPCR Master Mix (no ROX) (Thermo Scientific, USA), 0.5 µl of primer-probe mix (6 µl of 100nM forv. primer, 6 µl of 100nM rev. primer, 6 µl of probe and 100 µl of dd water), 1 µl of DNA samples. This method was used as an additional check of the tested patients with low levels of TRECs and/or KRECs. DNA sample with NBS was used as a positive control, DNA samples with normal level of TRECs/KRECs were used as a negative control, deionized water was used as a no template control. The results of 2 methods were quite similar.

The number of TREC and KREC copies per 10^6^ cells was calculated using the following formula (31):


$$\frac{1,000,000\;х\text{ mean SQ }\left(\text{TRECs or KRECs}\right)}{\text{Mean SQ }\left(\text{Albumin}\right)/2}$$

As a positive control, we used blood DNA samples of 65 patients with confirmed genetic and immunological diagnosis of IEI: 25 samples from the patients with ataxia-telangiectasia, 37 samples from patients with NBS (homozygous for the c.657del5 mutation of the NBN gene), one sample from a patient with XLA and 2 samples from the patients with SCID (JAK3 deficiency and DCLRE1C (Artemis) deficiency).

In cases where new sample test was needed, we collected additional information about the mother’s history and treatment during pregnancy, the newborn’s clinical condition, medicines taken (antibiotics, steroids), and laboratoty tests results.

### Statistical analysis

Statistical analysis were performed using STATISTICA 10. To compare continuous variables between the groups we used the Mann**–**Whitney test and the Kruskal**–**Wallis test. Qualitative variables are shown as absolute frequencies and percentages. Quantitative variables were tested using Kolmogorov**–**Smirnov test or Shapiro**–**Wilk test for normal distribution and are expressed as median and interquartile range (IQR), when appropriate. For quantitative variables the Mann–Whitney test were performed. P-values of <0.05 were considered as statistically significant.

### Definition and interpretation of the results

To avoid misunderstanding in terminology, we used published recommendations for uniform definitions in newborn screening for SCID (32). TREC copies above cut-off were determined as a *normal value*. Concordantly, TRECs below cut-off and without DNA ampflification failure were designated as an *abnormal value*. In the latter category, we distinguish an *urgent abnormal value* when TRECs were absent or very low (<100 copies per 10^6^ cells). In the case of DNA amplification failure the test result was considered as *incomplete*. DNA amplification failure was defined when albumin gene amplification curve was the same as NTC or in the case of PCR inhibition (number of amplification cycles for albumin was over 29). Usually, number of amplification cycles for albumin was in range of 24-28 in all samples. Repeated RT-PCR-assay from the same newborn screening card was defined as a *retest*. If a new sample was requested for TREC analysis, it was defined as a *new sample test*. In the case of abnormal TRECs value after new sample testing, the newborn was recall for exmination to confirm or to rule out the diagnosis. We define this event as a *referral.* The same was applied to the evaluation of thr results of the KRECs analysis.

**Figure 1:**
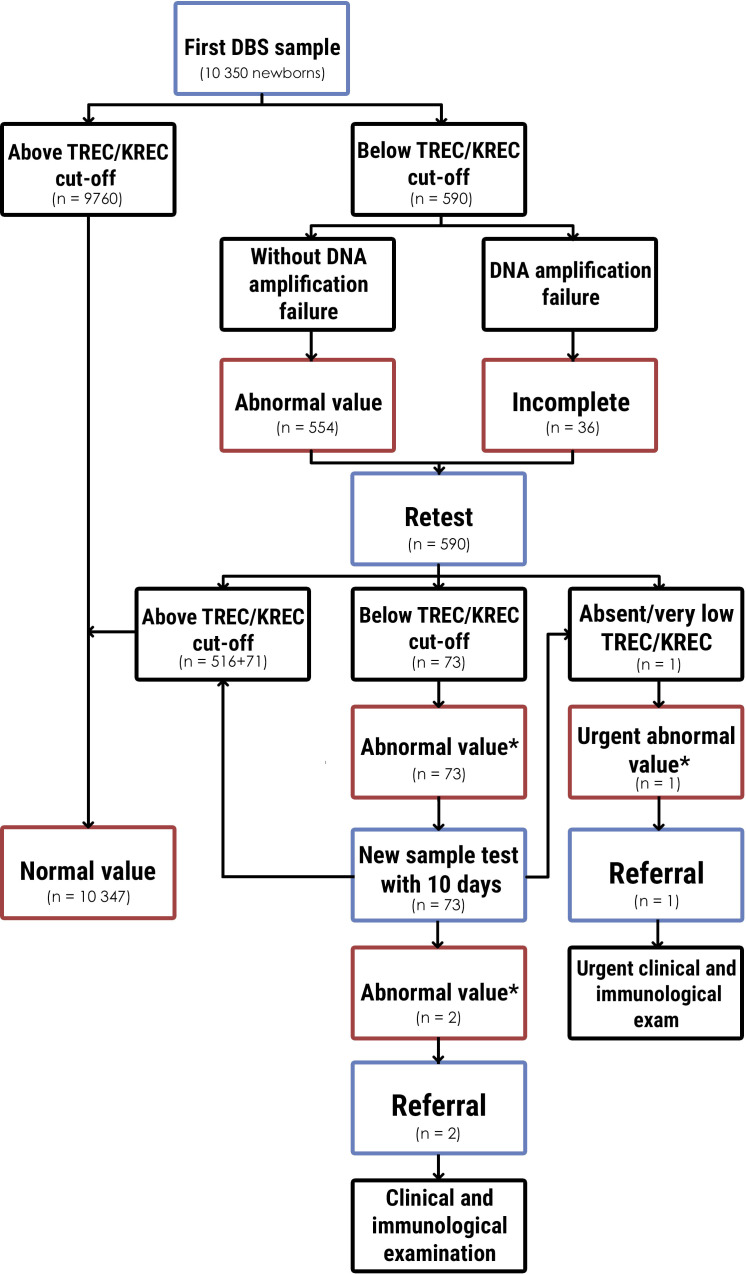
Newborn screening diagnostic algorithm for determination SCID and T- and B-lymphopenia and results. * - below TREC/KREC cut-off without DNA amplification failure.

SCID newborn screening diagnostic decision algorithm is demonstrated in **Figure 1**. To decrease false positive results and reduce parental stress and anxiety (33), we did a retest in the case of an abnormal value or incomplete results. If the retest result was also abnormal, the mother with a child were invited by a phone call to come for a new sample test (second sample) within 10 days. When a retest result was urgent abnormal in two tests from the first sample, the child was immediately referred for diagnosis confirmation to the Regional Children’s Hospital. The same algorithm was used with the new sample test. In the case of an abmormal value of the new sample test, the child was reffered to confirm the diagnosis. The patient underwent full clinical examination with an emphasis on detecting signs of immune deficiency, dysmorfic features and family history. Laboratory examination included complete blood count (CBC) with differential, lymphocyte subpopulation detected by cytometry assay, and immunoglobulins levels. Cytogenetic tests (karyotyping) and genetic testing (Next Generation Sequencing) were planned after clinical and immunological evaluation.

The main goal of this screening program was to identify newborns with SCID (defined as CD3 below 300 cells/µL). The second target was to identify newborns with non-SCID T- lympopenia and B-lympopenia, including combined immunodeficiency (CID), XLA, secondary immunodeficiencies, and other syndroms with T-cell impairment (15, 34, 35).

False-positive results were defined as abnormal TRECs and/or KRECs value in absence of SCID, XLA or other explanation of T- and/or B-lymphopenia.

## Results

**Figure 2:**
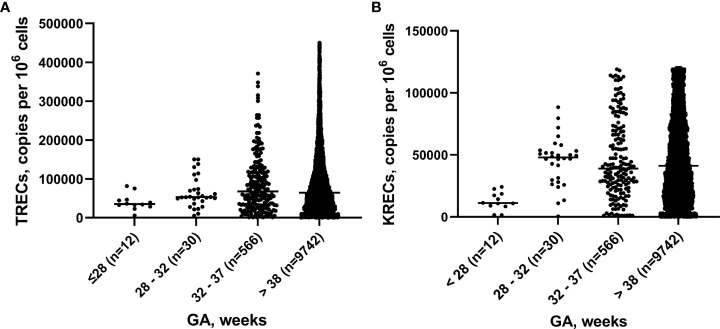
TRECs **(A)** and KRECs **(B)** (copies per 10^6^ cells) in newborns depending on gestation age (GA).

**Figure 3:**
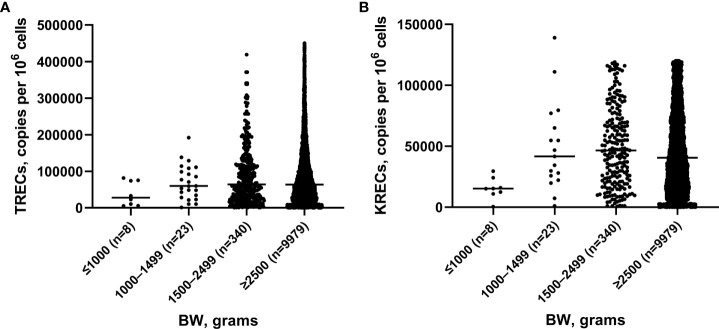
TRECs **(A)** and KRECs **(B)** (copies per 10^6^ cells) in newborns depending on birth weight (BW).

We analyzed 10,350 blood samples. The baseline characteristics of screened newborns are presented in **Table 1**. The gestational age of newborns ranged from 25 to 41 weeks, median – 38 weeks. There were 608 (5.87%) premature babies. The BW of the subjects ranged from 540 to 5,350 grams, median – 3,350 grams. The proportion of children with a BW of less than 2,500 grams was minor and amounted to 3.58% (371 newborns). TREC and KREC levels of screened newborns depending on GA and BW are presented in **Figures 2**, **3**.

**Table 1:** Baseline characteristics of the screened newborns.

Characteristic	n	%
Male/Female	5,279/5,071	51/49
Gestational age (GA)		
Extremely preterm (less than 28 weeks)	12	0.11
Very preterm (28 – 32 weeks)	30	0.29
Moderate preterm (32 – 37 weeks)	566	5.47
At term (GA ≥ 38 weeks)	9,742	94.13
Birth weight		
Less than 1,000 grams	8	0.08
1,000 – 1,499 grams	23	0.22
1,500 – 2,499 grams	340	3.29
≥ 2,500 grams	9,979	96.4

The Mann–Whitney test did not reveal significant differences between the median values of TREC levels in groups of children with different GA (p>0.05), whereas the median value of KRECs in extremely preterm newborn was significantly lower than in other groups with different GA (p=0.02, p=0.01, p=0.01, respectively). Meanwhile, the levels of both TRECs and KRECs was significantly lower in newborns weighing less than 1,000 grams compared to those weighing 1,500-2,499 grams (p=0.04). The level of KRECs was also significantly lower in the neonates weighing less than 1,000 grams compared to neonates weighing 1,000-1,499 grams and more than 2,500 grams (p=0.009, p=0.07, respectively).

### Determination of cut-off values

One of the challenges of a pilot screening program is determination of cut-off values (34). Since we used TREC/KREC assay to identify SCID and other T- and B-lymphopenia for the first time, it was important for us not to miss any newborns with these conditions.

At the first stage, a cut-off level of below 5,000 copies per 10^6^ cells for TREC and 5,000 copies per 10^6^ cells for KREC was applied considering referral values proposed in the published protocol (31). In total, 4,833 newborns were screened at this stage. Among them 366 (7.6%) required a retest and 45 (0.9%) required a new sample test (**Table 2**). One urgent abnormal value after retest was detected (TRECs – 0). Two mothers refused to take a new sample test. One child had an abnormal value (only TRECs) in the new sample test and was referred for clinical and laboratory examination. IEI was not confirmed in this child, however possible other reasons of TRECs/KRECs below the cut-off was suggested (**Table 3**).

**Table 2:** Number of newborns, retests from the first DBS, and recalls in the study population.

	I period	II period	Тotal
	5,000 cut-off	2,000 cut-off	
Newborns	4,833	5,517	10,350
Retest	366 (7.6%)	224 (4.1%)	590 (5.8%)
Referral (urgent abnormal value)	1 (0.02%)	0	1 (0.01%)
New sample test	45 (0.9%)	28 (0.5%)	73 (0.7%)
Referral to confirm/rule out the diagnosis	1 (0.02%)	1 (0.02%)	2 (0.02%)

**Table 3:** Characteristics of patients with abnormal value (urgent abnormal value and abnormal value in new sample test).

N	TRECs/KRECs, copies per 10^6^ cells	Diagnosis	Lymphocytes, cells/µL (%)	CD3, cells/µL (%)	CD4/CD8, cells/µL(%)	CD19,cells/µL (%)	IgA/IgM/IgG, g/l	Outcome
1.	0/31,200	CID (T^low^B+NK+) Unknown genetic cause	700-1,300 (13-17)	520 (39.8)	260/250 (20/19)	620 (47)	<0.15 /0.62 /3.7	Died 2 mos (COVID-19, pneumonia, venous thrombosis)
2.	4,010/16,300	Prematurity (GA–33 weeks), BW – 2300g. Transient lymphopenia	1,680-3,880 (12-73)	2,285 (59)	1,369/861 (35/22)	1261 (32)	na	Alive
3.	129,000/723	Mother – threatened abortion, polyhydramnios, progesterone –long period, antibiotic.	5,390 (64)	4,086 (76)	2,317/1,488 (43/28)	1,003 (18.6)	0.22 /0.44 /5.17	Alive

CID, combined immunodeficiency; GA, gestational age; BW, birth weight.

Taking into account a high rate of retests and new sample tests in addition to the results of our study of TREC/KREC levels in patient with ataxia-teleangiectasia (AT) (36), as well as the potential for high parental stress and anxiety, we reduced the cut-off threshshold to 2,000 copies per 10^6^ cells for TRECs and KRECs. Another 5,517 newborns were screened during this stage. The rate of retest decreased to 4.1%, and proportion of abnormal value results declined to 0.5%. One child was referred to confim or rule out immunodeficiency (**Table 2**).

Characteristics of the patients with the urgent abnormal value and abnormal value in a new sample test results are presented in **Table 3**. Of this group, the first patient had an urgent abnormal value after retest (0 TREC twice). Determination of the lymphocyte subpopulations by flow cytometry confirmed the T-cell deficiency and the diagnosis of CID (T^low^B+NK+). Unfortunately, the patient died at the age of 2 months due to COVID-19, complicated by pneumonia, venous thrombosis and progressive multiple organ failure. The amount of available biological material did not allow a further in-depth genetic research to determine the presence of specific mutations.

### Samples from patients with known IEI (controls)

**Figure 4:**
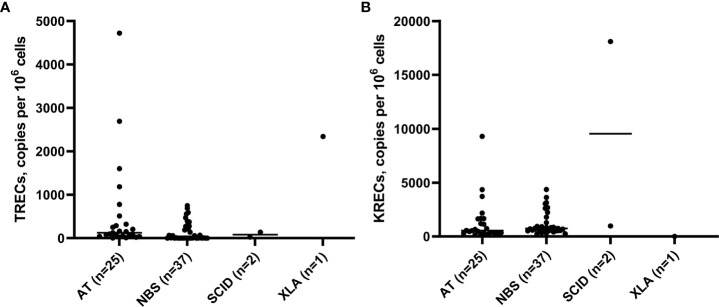
TRECs **(A)** and KRECs **(B)** (copies per 10^6^ cells) in a control group (patients with AT, NBS, SCID (JAK3 deficiency and DCLRE1C deficiency), and XLA.

To assess the quality and sensitivity of TREC/KREC assay, we performed it on blood samples of the patients with confirmed immunodeficiencies. The control group consisted of 25 patients with AT, 37 patients with NBS, 1 patient with XLA and 2 patients with SCID, i.e. one with JAK3 deficiency (T-B+) and one with DCLRE1C (Artemis) deficiency (T-B^low^). The average age of patients with AT at the time of examination was 8.45 ± 2.74 years, ranging from 3 to 14 years. The average age of patients with NBS was 4.5 years, ranging from 1 month to 13 years. TREC levels in all patients with AT and NBS were below 5,000 copies per 10^6^ cells, whereas KRECs levels were below 10,000 copies per 10^6^ cells in patients with AT (**Figure 4**). In the patients with NBS, 100% sensitivity of TREC and KREC values were 2,000 copies per 10^6^ cells (**Table 4**). TREC levels in patients with SCID were less than 200 copies per 10^6^ cells: 138 in the patient with JAK3 deficiency and 25 in the patient with DCLRE1C deficiency. Accordingly, KRECs were absent in the patient with XLA and were less than 1,000 copies per 10^6^ cells in the patient with DCLRE1C (Artemis) deficiency. Albumin level was normal in all controls included to the study.

**Table 4:** Sensitivity of method depending on TREC/KREC cut-offs levels in patients of controls (AT and NBS patients).

Сut-off, copies per 10^6^ cells	Sensitivity					
	TREC			KREC		
	AT (n=25)	NBS (n=37)	AT+NBS (n=62)	AT (n=25)	NBS (n=37)	AT+NBS (n=62)
10000	25 (100%)	37 (100%)	62 (100%)	25 (100%)	37 (100%)	62 (100%)
5000	25 (100%)	37 (100%)	62 (100%)	24 (96%)	37 (100%)	61(98.4%)
4000	24 (96%)	37 (100%)	61(98.4%)	23 (92%)	37 (100%)	60 (96.8%)
3000	24 (96%)	37 (100%)	61 (98.4%)	22 (88%)	37 (100%)	59 (95.2%)
2000	**22 (88%)**	**37 (100%)**	**59 (95.2%)**	**21 (84%)**	**37 (100%)**	**58 (93.5%)**
1000	21 (84%)	37 (100%)	58 (93.5%)	15 (60%)	28 (75.7%)	43 (69.4%)
500	19 (76%)	26 (70.3%)	45 (72.6%)	12 (48%)	5 (13.5%)	17 (27.4%)
100	11 (44%)	16 (43.2%)	27 (43.5%)	0	0	0

AT, ataxia-telangiectasia; NBS, Nijmegen breakage syndrome. The sensitivity of TRECs and KRECs with cut-off 2000 copies per 106cells is highlighted in bold.

## Discussion

Today, the most promising approach in Europe is to use the TREC/KREC/ACTB triplex assay for newborn screening to detect severe T- and B-cell lymphopenias. This method is included in the national programs of many European countries, and some of them have already launched pilot projects (6, 9). Meanwhile, the USA, Canada, Israel, Taiwan, Saudi Arabia, and a number of other countries, have implemented TREC assay for SCID screening (11–14, 17). Among the countries of Central and Eastern Europe, the first population-based screening for T- and B-lymphopenia began in 2017 in the Polish-German transborder area (15). We report about the first pilot study of newborn screening for SCID in Ukraine using TREC/KREC assay.

It should be noted that currently two approaches are being used: DNA isolation followed by RT-qPCR (TREC/KREC/ACTB-assay) and commercial kits such as EnLite™ TREC kit for newborns from PerkinElmer (USA) and SCREEN newborn screening kit ID ImmunoIVD (Sweden) (**Table 5**). The EnLite Neonatal TREC test is intended for the semiquantitative multiplex determination of TREC and β-actin. It involves perforating DBS samples with a 1.5 mm punch head. The SCREEN-ID ImmunoIVD allows the quantification of TREC and/or KREC, β-actin as a quality control marker by quantitative real-time multiplex PCR (qPCR) using the ordinary 3.2 mm DBS. The kit includes all the necessary reagents pre-packaged in a set of elution and qPCR plates and requires only two pipetting steps. This technique allows to determine either only TRECs, or TRECs and KRECs simultaneously (18). Cut-off levels for these markers mostly depend on the chosen method (**Table 4**). The TRECs cut-off for the EnLite Neonatal TREC assay to discriminate screen-positive samples based on the manufacturer’s recommendations is 30 copies/μl. For the SCREEN-ID kit, the TRECs cut-off according to manufacturer’s recommendation is 6 copies/μl, while for KRECs it is 4 copies/μl. However, both manufacturers still recommend a pilot study with a large number of samples to establish a desired cut-off value based on a normal population distribution (18). Even though we have initially planned to use a commercial kit for this pilot project, this was hindered by the COVID-19 pandemic, the onset of which coincided with the start of the project. Since the restrictions caused difficulties with staff training and purchase of kits, we started to determine TRECs and KRECs in neonatal DBS using an RT-PCR adapted method, followed by the analysis of melting curves. Therefore, it was crucially important for us to establish the optimal cut-off values, which would, on the one hand, allow to capture all instances of SCID, XLA, and other diseases that present with severe T- and B-lymphopenia, and, on the other hand, to avoid a large number of false positive outcomes to reduce the potential for parental stress and anxiety.

**Table 5:** Comparison of the SCID newborn screening results in different studies.

Study	Newborns, n	Cutoff TREC/KREC	Sample processing	Retest	Referral(after 1st retest)	New sample test	Referral	Rate of the referral	SCID detected
Verbsky JW. et al. (Wisconsin, USA, 2012) (11)	207,696	25 TRECs/μl -1^st^ year; 40 TRECs/μl-next year	RT-qPCR of TRECs and β-actin	na	63 (0.03%)	386 (0.19%) including abnormal in pretern and inconclusive)	9 (0.004%)	1: 2,884	5
Giżewska M. et al. (Poland-German, 2020) (15)	44,287	< 6/<4 copies/µL	Commercial kit (ImmunoIVD, Sweden)	321 (0.72%)	7 (0.02%)	68 (0.15%)	1 (0.002%)	1: 5,366	1 + 1(CID)
de Felipe B. et al. (Seville, Spaine, 2016) (16)	5,160	< 6/<4 copies/punch	RT-qPCR (TRECs/KRECs/ACTB-assay)	77 (1.5%)	na	10 (0.19%)	5 (0.1%)	1: 1,032	0
Argudo-Ramírez A. et al. (Catalonia, Spain, 2019) (24)	130,903	≤ 34 copies/µL	Commercial kit (PerkinElmer, Finland)	3108 (2.4%)	12 (0.01%)	304 (0.2%)	18 (0.01%)	1: 4,363	1
Barbaro M. et al. (Sweden, 2017) (35)	58,834	Last – 10/6 copies/3.2 mm punch	RT-qPCR (TRECs/KRECs/ACTB-assay)	572 (0.97%)	na	64 (0.11%)	3 (0.005%)	1: 20,000	1
Thomas C. et al. (French, 2019) (23)	190,517	≤ 34 copies/µL	Commercial kit (PerkinElmer, Finland)	na	139 (0.07%)	291 (0.15%)	26 (0.014%)	1: 1,154	3 + 3 leaky SCID
Chien YH. et al. (Taiwan, 2015) (14)	106,391	< 40 TRECs/μl	RT-qPCR of TRECs	na	5 (0.005%)	432 (0.4%)	19 (0.018%)	1: 4,433	2
Rechavi E. et al. (Israel, 2017) (34)	177,277	From 36 to 23 copies/blood sport	Commercial kit (PerkinElmer, Finland)	4.24% (for 36) 0.95% (for 23)	na	561 (0.3%)	46 (0.02%)	1: 3,853	8
Adams SP. et al. (UK, 2014) (37)	5,099	< 40 TRECs/μl	Commercial kit (PerkinElmer, Finland)	209 (4.10%)	na	51 (1.0%)	–	na	18 (control)
Al-Mousa H. et al. (Saudi Arabia, 2018) (17)	8,718	< 36 copies/μl	Commercial kit (PerkinElmer, Finland)	315 (3.6%)	16 (0.18%)	–	–	1: 545	3
Blom M. et al. (Netherland, 2018) (18)	1,272	30 copies/μL/ 6 copies/μL	Commercial kit (PerkinElmer, Finland/Immuno IVD) Sweden) and	na	na	38 (3.0%)/ 5 (0.39%)	–	na	
This study	10,350	<5,000 copies/per 10^6^cells -1^st^ stage; <2,000 copies/per 10^6^cells – next stage	RT-qPCR (TRECs/KRECs/albumin-assay)	590 (5.8%)	1 (0.01%)	73 (0.71%)	2 (0.02%)	1: 3,450	1 (CID)

Therefore, at the first stage, we decided on the cut-off of 5,000 copies per 10^6^ cells in order not to miss cases of other immunodeficiency conditions that present with T- and B-lymphopenia. At this stage, we detected one case of CID with urgent ubnormal value of TRECs (twise 0) that was confirmed by immunological examination (T^low^B+NK+). However, with this cut-off value, the retest proportion was high (7.6%) in comparison to other studies (**Table 5**) where percentage ranged from 0.72% (15) to 4.24% (34). New sample tests were needed for 45 (0.93%) newborns, which was also higher than reported in other studies (15, 16, 23, 24, 35), although it was comparable to the results of individual pilot studies (37). Only one child required a referral for further immunological examination based on the results of the new sample test. This was a premature baby (GA 34 weeks), with BW of 2,300 grams and TRECs level 4,010 copies per 10^6^ cells. The child was diagnosed with transient lymphopenia and was followed up more than one year, during which period severe infections were not observed.

Since the parents expressed their concern in the instances of having to prefornm a repeat blood stain, and taking into account a previous study on TRECs/KRECs in patients of the control group, the cut-off level at the second stage was reduced to 2,000 copies per 10^6^ cells, which made it possible to reduce the number of retests and new sample tests almost two-fold and bring our outome indicators closer to the results of other published studies (17, 24, 34, 37).

As reported by other researchers, T- and/or B-cell lymphopenia in newborns is most often a result of maternal immunosuppression, prematurity, or congenital heart defects (15). Lower levels of TRECs in preterms were first pointed out to in a USA study (12). In Sweden, 40% of babies with lymphopenia were born prematurely (<37 weeks of gestation) (26), although no direct correlation with decreasing GA was observed (35), and the majority of results in preterms were above cut-off values. These results are comparable with our results, since we did not find a significant difference between the TREC median indicators in groups of children with different GA. Additionally, our study showed the correlation between the levels of TRECs and KRECs and the body weight of newborns; these levels were especially low in children with a weight of less than 1,000 grams. Other studies have shown low levels of TRECs/KRECs in twins and triplets (35). This may be related to both prematurity and low birth weight, a question which requires further investigation.

To reduce the need for new sample tests, researches use different strategies (33). While some screening programs set up different cut-off levels for full-term and premature babies (24), others contend that cut-off values require no change for preterm newborns despite a higher rate of retest in this cohort (23). Finally, some researches use three retests in the instances of abnormal TREC values and then take into account 2 out 3 results (24).

In our study, the referral rate, including caused by the urgent abnormal value was 0.03%, or 1 in 3,450 screened newborns. It is comparable with other published results (**Table 5**), where this number ranged from 1:545 in Saudi Arabia (17) to 1:20,000 in Sweden (35).

The results of TREC/KREC assay in patients of the control group showed that its sensitivity for detecting severe combined immunodeficiencies, in particular AT and NBS, is 95.2% for TRECs and 93.5% for KRECs, with the cut-off level of 2,000 copies per 10^6^ cells. Meanwhile, for the patients with SCID, TREC levels were less than 1,000 copies per 10^6^ cells, and as low as 138 in the patient with JAK3 deficiency and 25 in patient with DCLRE1C deficiency. KREC in the patient with XLA was 0.

Thus, TREC/KREC assay allows to detect both SCID and other CIDs that overlap with T- and B-lymphopenia. In the future, to reduce the anxiety of parents, cut down the number of retests, and therefore the cost of screening, the cut-off level can be lowered to 1,000, which will allow to capture SCID and effectively detect conditions accompanied by T- and B-lymphopenia.

Our study also showed a higher efficiency and sensitivity of TRECs detection, therefore, for the further implementation of screening in Ukraine, especially in the context of limited resources related to the war and COVID-19, it is possible to use only TREC assay to screen for SCID.

The RT-PCR technique used in the study allows establishing the number of TRECs and KRECs relative to the number of copies of the albumin gene, unlike commercial kits that provide data in copies per microliter or copies per punch. The use of commercial kits to determine TRECs and KRECs has the advantage of uniformity, but at the same time it is much more expensive.

Despite a number of advantages for the simultaneous use of TREC/KREC assay compared to the use of TRECs alone, in particular for the detection of congenital B -cells defects, as well as late onset ADA deficiency (22), currently there is no consensus regarding the use KRECs assay in newborn screening and compliance of the IEI diagnosis associated with only B-lymphopenia with the general principles of newborn screening (6). In our study, only one child had a reduced level of KRECs together with a normal value of TRECs in a new sample test. The boy was born full-term (39 weeks) by cesarean section, weighing 3,750 g. During pregnancy, the mother had a threat of abortion, polyhydramnios and for a long time took progesterone drugs. The mother also recalled taking antibiotics during pregnancy. No other previously described causes of low KREC values were observed in the child. It is noted that B-lymphocytes are more sensitive to drug-induced apoptosis than T-lymphocytes, although, as described in the literature, these effects were associated with azathioprine (35). Further immunological examination of the boy did not reveal any pathological changes. Thus, the result was considered a false positive. The child was followed for 1,5 years during whih time he had 3 episodes of acute gastroenteritis. The Swedish study did not identify any patients with primary immunodeficiency based solely on low KREC levels during the three-year screening period, however the researchers still suggest that the triple assay is most suitable for newborn SCID screening to identify infants with NBS and XLA (26) that is especially relevant in patients of Eastern Slav origin (38).

It is worth underscoring that the newborn screening is only the first step on the way to a diagnosis, and in some instances a definitive diagnosis might be not achieved (6). A successful screening program has to include a system of measures, such as genetic counseling of the family, repeat testing, additional laboratory and instrumental studies, full support of the sick child and constant monitoring of their condition.

### Strengths and limitation of the study

The strength of this pilot study is the determination of T- and B-lymphopenia using TREC and KREC assay by RT-PCR in neonatal dry blood spots. Another positive aspect is modification of the technique using melting curves analysis, which made it possible to avoid a large number of false negative results. In general, the obtained results were comparable to the data of other studies (**Table 5**). The selected cut-off levels for TREC and KREC were incrementally optimized to avoid unnecessary sampling and testing associated with unjustified additional costs. The authors also followed-up children with low TRECs and KRECs from 2 months to 2 years of age to determine the impact of lymphopenia at birth on subsequent morbidity in children.

A limitation of the study is a small group of screened newborns (10,350), covering only one region of Ukraine, which did not make it possible to detect a larger number of SCID and establish the prevalence of the disease, since its average frequency is 1 per 50-100 thousand newborns (5, 6). The TREC and KREC assay using proposed RT-PCR method for detecting T- and B-lymphopenia needs further standardization to successfully implement it in the newborn screening program for SCID. The small number of premature infants in this pilot study may have been the reason for no significant difference in the number of TRECs and KRECs in premature infants compared to full-term infants, as noted in other studies (34, 39). However, this number of screened infants is acceptable for a pilot study, which has the goal of testing the methodology and establish cut-offs, optimize the algorithm to avoid both false positive and false negative results.

## Conclusions

The recognition of patient with CID in this pilot study with involving of 10,350 newborns and the results of 65 tested patients of control group with confirmed inborn errors of immunity has shown that the tested method for determination of TRECs and KRECs by RT-PCR followed by analysis of melting curves in neonatal dry blood spots is effective for the detection of severe T- and B-lymphopenia and can be used in newborn screening for SCID in Ukraine. Although determination of TREC in newborn screening for SCID has proven high sensitivity, it should be stressed that in order not to miss any other types of PID as agammaglobulinemia, Nijmegen breakage syndrome, AT or DiGeorge syndrome, as well as late-onset ADA SCID, newborn screening for inborn errors of immunity in the form of triple assay (including TREC and KREC) is optimal.

## Data availability statement

The data analyzed in this study is subject to the following licenses/restrictions: The datasets of this study are available on request from the corresponding author. Requests to access these datasets should be directed to boyarchuk@tdmu.edu.ua.

## Ethics statement

The study was approved by the I.Horbachevsky Ternopil National Medical University Ethics Committee (Minutes numero 55 from November 4, 2019). Written informed consent to participate in this study was provided by the participants’ legal guardian/next of kin.

## Authors contributions

OB and HM designed and concept of the manuscript. OB, NY, and TH designed the concept of the pilot study project. NY, OS, IC, TH, LV, and MK were responsible for sample collection and logistic. VK, IS, and HM did the laboratory work. NY provided statistical analysis. OB, NY, VK, and HM analyzed and interpreted the data. OB, NY, and VK prepared figures and tables. OB, OS, and LV collected the relevant information and references. OB, OS, LV, and HM wrote the manuscript with contribution from all co-authors. All authors read, critically reviewed and approved the final version.

## Funding

The study was financed by the Ministry of Health of Ukraine with funds from the state budget, project title “Pilot study on neonatal screening of primary immunodeficiencies by the method of TRECs and KRECs for the determination of T- and B-lymphopenias”, state registration number 0120U104282, implementation period - 2020-2022.

## Acknowledgments

We would like to thank doctors of the Western-Ukrainian Specialized Children’s Medical Center, Lviv, Ukraine: Larysa Kostyuchenko, Yaryna Romanyshyn and of the Ivano-Frankivsk Regional Children’s Hospital, Ivano-Frankivsk, Ukraine: Olha Fedynska, Monika Makyan for their help in the enrollment of the control group. We wish to express our gratitude to all neonatologists of Ternopil region for their help in sample collection. We appreciate for providing the training on TRECs and KRECs standards by Ekaterina Polyakova from the Belarusian Research Center for Pediatric Oncology, Hematology and Immunology, Minsk Region, Belarus; to Dr. Linda Gailite from Scientific Laboratory of Molecular Genetics, Riga Stradiņs University, Latvia for reagents supply on the last stage of the study.

## Conflict of interest

Authors VK, IS, and HM were employed by Scientific Medical Genetic Center LeoGENE, LTD, Lviv, Ukraine.

The authors declare that the research was conducted in the absence of any commercial or financial relationships that could be construed as a potential conflict of interest.

## Publisher’s note

All claims expressed in this article are solely those of the authors and do not necessarily represent those of their affiliated organizations, or those of the publisher, the editors and the reviewers. Any product that may be evaluated in this article, or claim that may be made by its manufacturer, is not guaranteed or endorsed by the publisher.
